# Physical activity maintenance among young adult cancer survivors in an mHealth intervention: Twelve‐month outcomes from the IMPACT randomized controlled trial

**DOI:** 10.1002/cam4.6238

**Published:** 2023-06-14

**Authors:** Carmina G. Valle, Molly A. Diamond, Hillary M. Heiling, Allison M. Deal, Derek P. Hales, Brooke T. Nezami, Jessica Gokee LaRose, Christine M. Rini, Bernardine M. Pinto, Deborah F. Tate

**Affiliations:** ^1^ Gillings School of Global Public Health University of North Carolina at Chapel Hill Chapel Hill North Carolina USA; ^2^ Lineberger Comprehensive Cancer Center University of North Carolina at Chapel Hill Chapel Hill North Carolina USA; ^3^ Department of Health Behavior and Policy, School of Medicine Virginia Commonwealth University Richmond Virginia USA; ^4^ Department of Medical Social Sciences, Feinberg School of Medicine Northwestern University Chicago Illinois USA; ^5^ Robert H. Lurie Comprehensive Cancer Center of Northwestern University Chicago Illinois USA; ^6^ College of Nursing University of South Carolina Columbia South Carolina USA

**Keywords:** activity tracker, cancer survivors, digital health, intervention, maintenance, mHealth, physical activity, social media, young adults

## Abstract

**Background:**

Most physical activity (PA) interventions in young adult cancer survivors (YACS) have focused on short‐term outcomes without evaluating longer‐term outcomes and PA maintenance. This study examined the effects of an mHealth PA intervention at 12 months, after 6 months of tapered contacts, relative to a self‐help group among 280 YACS.

**Methods:**

YACS participated in a 12‐month randomized trial that compared self‐help and intervention groups. All participants received an activity tracker, smart scale, individual videochat session, and access to a condition‐specific Facebook group. Intervention participants also received lessons, tailored feedback, adaptive goal setting, text messages, and Facebook prompts for 6 months, followed by tapered contacts. Accelerometer‐measured and self‐reported PA (total [primary outcome], moderate‐to‐vigorous [MVPA], light, steps, sedentary behaviors) were collected at baseline, 6, and 12 months. Generalized estimating equation analyses evaluated group effects on outcomes from baseline to 12 months.

**Results:**

From baseline to 12 months, there were no between‐ or within‐group differences in accelerometer‐measured total PA min/week, while increases in self‐reported total PA were greater in the intervention versus self‐help group (mean difference = +55.8 min/week [95% CI, 6.0–105.6], *p* = 0.028). Over 12 months, both groups increased accelerometer‐measured MVPA (intervention: +22.5 min/week [95% CI, 8.8–36.2] vs. self‐help: +13.9 min/week [95% CI, 3.0–24.9]; *p* = 0.34), with no between‐group differences. Both groups maintained accelerometer‐measured and self‐reported PA (total, MVPA) from 6 to 12 months. At 12 months, more intervention participants reported meeting national PA guidelines than self‐help participants (47.9% vs. 33.1%, RR = 1.45, *p* = 0.02).

**Conclusion:**

The intervention was not more effective than the self‐help group at increasing accelerometer‐measured total PA over 12 months. Both groups maintained PA from 6 to 12 months. Digital approaches have potential for promoting sustained PA participation in YACS, but additional research is needed to identify what strategies work for whom, and under what conditions.

## Graphical abstract

The IMproving Physical Activity after Cancer Treatment trial tested whether an mHealth physical activity intervention for young adult cancer survivors was more effective than a self‐help group at improving total amount of physical activity and other health outcomes over 12 months among 280 participants around the United States. The intervention was not more effective at increasing accelerometer‐measured total physical activity over 12 months relative to a self‐help group that only received digital tools. However, increases in self‐reported total physical activity were significantly greater in the intervention group, and both groups maintained levels of accelerometer‐measured total and moderate‐to‐vigorous physical activity from 6 to 12 months.

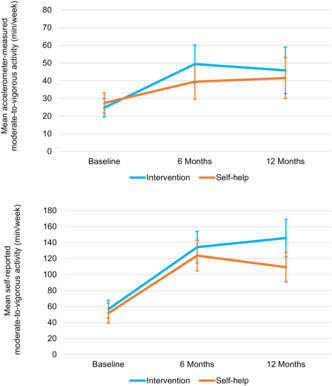

## 1 INTRODUCTION

Young adult cancer survivors (YACS), diagnosed between ages 18–39, are at increased risk for morbidity and developing chronic disease,^1^, ^2^ and may face decades at risk for late effects.^3^ Physical activity (PA) confers several benefits for cancer survivors, including improvements in quality of life, fatigue, anxiety, depressive symptoms, and physical function, and it may reduce risk for chronic disease, long‐term, and late effects.^4^ Yet, the majority of YACS are not meeting national PA guidelines for cancer survivors.^5^, ^6^ As cancer survivors may be challenged to meet these guidelines and evidence accumulates on the positive health effects of light PA,^7^, ^8^ promoting total PA may be beneficial. Despite high interest in PA guidance and support among young adults with cancer,^9^, ^10^ few interventions have addressed the unique needs of this population.^11^, ^12^

Previous digital PA interventions have shown potential among YACS, but most studies are limited by small sample sizes and short duration (8–12 weeks).^12^, ^13^ For cancer survivors to experience the benefits of PA interventions, sustained adherence is needed;^14^ yet, they face challenges engaging in and maintaining PA after treatment.^14^, ^15^, ^16^ Few randomized trials of PA behavior change interventions among cancer survivors report long‐term outcomes at 12 months or beyond,^17^, ^18^, ^19^, ^20^ and previous systematic reviews have identified the need for more studies to evaluate longer‐term PA behavior change and maintenance of PA outcomes following interventions among cancer survivors.^19^, ^21^, ^22^, ^23^, ^24^ A recent meta‐analysis of lifestyle interventions for cancer survivors found that interventions have maintained improvements in self‐reported levels of moderate‐to‐vigorous intensity PA (MVPA) at 3 months post‐intervention,^19^ but fewer have demonstrated PA maintenance at 6 months or longer post‐intervention.^19^, ^24^ Evaluation of longer‐term total PA and maintenance of behavior change following digital interventions could guide the development of more optimal strategies to promote longer adherence, and afford rationale for wider dissemination in community and clinical settings.^17^ To our knowledge, no studies have examined longer‐term PA outcomes beyond 6 months or maintenance of PA at 6 months post‐intervention among YACS.

We recently completed the IMPACT trial, the longest randomized controlled trial of a PA intervention among YACS to date.^25^, ^26^ Given their desire for technology‐delivered interventions, all participants received an individual videochat session and digital tools, including an activity tracker, smart scale, and access to a closed Facebook group for peer support. We tested an mHealth intervention that provided theory‐ and evidence‐based enhancements wrapped around the digital tools, including adaptive goal setting, tailored feedback, and text messages, compared with a self‐help group that received digital tools alone. The intervention was designed to promote total PA and to enhance our earlier Facebook‐based intervention that resulted in greater increases in total and light PA compared with a self‐help group over 12 weeks.^27^ After a 6‐month intervention, participants had continued but tapered contacts, including access to the intervention Facebook group and intervention website, where they received bimonthly refresher lessons and tailored feedback and could set weekly goals to support increasing PA over time. We previously reported in our publication of PA outcomes at 6 months (i.e., primary time point post‐intervention) that there were no significant between‐group differences in accelerometer‐measured total PA min/week (primary outcome).^26^ Both intervention and self‐help groups increased MVPA (secondary outcome) over 6 months, with a trend indicating the intervention might be more favorable. The goal of the present study was to evaluate the effects of the intervention relative to the self‐help group on longer‐term changes in total PA min/week (accelerometer‐measured and self‐report) at 12 months. We hypothesized, a priori, that YACS in the intervention group would demonstrate greater increases in total PA min/week from baseline to 12 months and better maintenance of changes in total PA from 6 to 12 months relative to those in the self‐help group. Secondary objectives were to compare the two groups on changes in MVPA and light PA min/week, steps per day, and sedentary behavior, and the proportion of participants meeting national guidelines of ≥150 min/week of MVPA at 12 months.^4^

## 2 METHODS

### 2.1 Study Design

The IMPACT trial was a 12‐month, 2‐arm randomized controlled trial of an mHealth intervention designed specifically for YACS compared with a self‐help condition. The study protocol and primary (total PA) and secondary (MVPA, light PA, steps, sedentary behaviors) outcomes at 6 months (primary time point) have been previously reported.^25^, ^26^ PA outcomes were assessed at baseline, 6, and 12 months. The current study reports on total PA, MVPA, light PA, steps, and sedentary behavior at 12 months—the same PA measures for which we previously published 6‐month outcomes.^26^

### 2.2 Participants and Sample

Participants were YACS, ages 18–39, who were post‐treatment, within 10 years of diagnosis, and not meeting PA recommendations of ≥150 min/week of MVPA (as measured by accelerometer). Details on participant recruitment and the study sample (*N* = 280) are described elsewhere.^25^, ^28^ All individuals provided informed consent prior to participation. Participants were 33.4 (SD = 4.8) years of age on average, with BMI of 30.1 (SD = 8.3), and 3.66 (SD = 2.41) years from their cancer diagnosis. Most participants were women (82%), non‐Hispanic White (77%), with college degrees (71%), health insurance (92.9%), and reported incomes ≥$60,000 (52.5%). Data were collected from 2018 to 2021, and analyses were conducted in 2022.

### 2.3 Procedures and study conditions

The intervention and self‐help conditions have been previously described in detail.^25^ Briefly, all participants received an individual videochat session (discussion of study procedures, current PA guidelines for cancer survivors, and the benefits of light PA) and digital tools (i.e., Fitbit activity tracker with companion mobile app, smart scale, access to a condition‐specific Facebook group).

#### 2.3.1 IMPACT intervention

Active intervention delivery to the intervention group occurred during months 1–6, with tapered contacts from months 7–12. Intervention components were designed using social cognitive theory^29^ as a guiding framework. They used strategies and behavior change techniques to promote behavioral capability, self‐regulation, self‐efficacy, and social support.^25^ In addition to the individual videochat session and digital tools, intervention participants also had access to a mobile website with adaptive PA goal recommendations (weekly in months 1–12), behavioral lessons (22 total, weekly in months 1–3, biweekly in months 4–6, one in months 8, 10, and 12), tailored feedback summaries (28 total, weekly in months 1–6, one in months 8, 10, and 12), and publicly available web resources. Additionally, the intervention provided text messages (i.e., 5 per week in months 1–6, 1 per week in months 7–12) and prompts to engage within the condition‐specific Facebook group (i.e., up to 5 per week in months 1–12).

#### 2.3.2 Self‐help

Along with the initial videochat session, self‐help participants had continued access to the digital tools for the duration of the study (i.e., months 1–12), including the condition‐specific Facebook group. Throughout months 1–12, study interventionists monitored Facebook group activity and posted when new cohorts of participants were added but did not encourage engagement with specific prompts or provide any additional contacts.

### 2.4 Measures

PA was measured at baseline, 6, and 12 months using accelerometers (ActiGraph GT3X+, Pensacola, FL) and self‐reported with a modified version of the Godin Leisure Time Exercise Questionnaire,^30^, ^31^ as prespecified in our protocol^25^ to facilitate comparisons with previous studies. Additional details on PA calculations are published elsewhere.^25^ Briefly, participants were asked to wear accelerometers for 7 days; data were considered valid if worn for ≥4 days, with at least one weekend day and ≥10 h of waking wear.^32^ Accelerometer‐measured PA outcomes were calculated from minutes of waking wear using standard cutpoints^33^, ^34^, ^35^ and bout counting algorithms^36^, ^37^ applied to vector magnitude estimates. Bout minutes (i.e., 10+ min) of total PA (primary outcome; sum of light, moderate, and vigorous PA), MVPA, and light PA were aggregated at the day and participant level for analyses, and bout min/week were calculated (i.e., (5 * weekday average) + (2 * weekend day average)). Self‐reported PA was calculated from participant responses to the modified Godin Leisure Time Exercise Questionnaire,^21^, ^22^ by multiplying reported frequency of engaging in light, moderate, and vigorous exercise by the reported average duration (in min) during a typical week. Steps/day and sedentary min/day were computed from sums of all waking minutes. Self‐reported weekday and weekend sedentary behaviors were measured using the Sedentary Behavior Questionnaire.^38^ Days that participants tracked activity (i.e., ≥200 steps/day on Fitbit), engagement within the Facebook group, and website logins were collected as process measures. Participants reported medical events and symptoms through online questionnaires at 3, 6, and 12 months or by initiating contact with study staff. Details on measures are published elsewhere.^25^

### 2.5 Statistical analyses

Descriptive analyses were conducted to summarize participants' baseline characteristics by condition. Fisher's exact tests compared the groups on reported medical events. Wilcoxon rank sum tests compared the groups on days of activity tracking and Facebook engagement. To evaluate intervention effects relative to the self‐help group, we used an intention‐to‐treat approach including data from all participants. We conducted repeated measures analyses using generalized estimating equation (GEE) analyses to compare the effects of the intervention and self‐help groups on total PA min/week over the course of the 12‐month study. Based on examination of spaghetti plots showing the trajectory of accelerometer‐measured and self‐reported activity over time from baseline to 6 to 12 months, it appeared that the general activity trends over time were often non‐linear. Therefore, time was treated as categorical to allow the longitudinal model to accommodate non‐linear trends over time. The longitudinal analyses modeled accelerometer‐measured and self‐reported PA outcomes (total, MVPA, light, steps, sedentary behaviors) by group, time as a 3‐level category (i.e., baseline as reference, month 6, and month 12), and the interaction between group and time. These models estimated activity changes over time from baseline to 12 months and 6 to 12 months within groups, as well as differences in changes over time from baseline to 12 months and 6 to 12 months between groups. As prespecified in the study protocol, adjusted models included education, time since diagnosis, age, and wear time (accelerometer‐measured outcomes only) as covariates.^25^ Sensitivity analyses examined total PA and MVPA outcomes among participants with complete data at all time points, and when removing outliers (i.e., defined as ≥3 SD from mean change over time). Additionally, modified Poisson models were used to model the binary outcome of meeting national PA guidelines (MVPA ≥150 min/week) at 12 months (accelerometer‐ and self‐reported measures) by group, adjusted for covariates; these models provide relative risk estimates. All analyses were conducted with SAS (v9.4).

## 3 RESULTS

### 3.1 Participant characteristics and adherence

**FIGURE 1:**
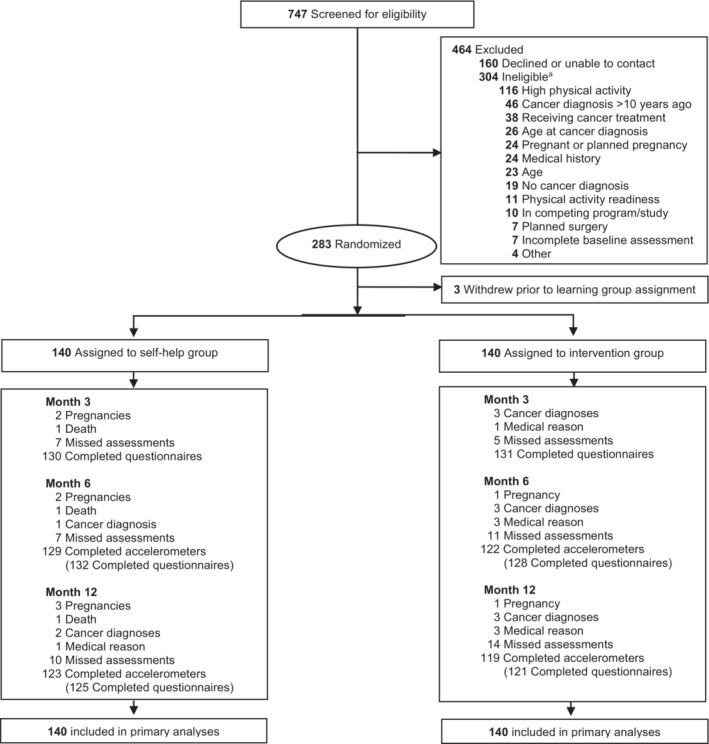
Consolidated standards of reporting trials diagram of participant flow. ^a^There may be >1 reason for ineligibility.

Of 280 participants randomized, 242 completed accelerometer assessments (86.4%) at 12 months, of which 236 (84.3%) provided valid accelerometer data (6 malfunctioning accelerometers); 246 (87.9%) completed valid self‐report PA questionnaires (Figure 1). Retention did not differ by group (*p*s >0.71) or baseline characteristics (*p*s >0.05), except that males were more likely to complete accelerometer assessments (*p* = 0.03) and participants who were not working were less likely to complete self‐report assessments (*p* = 0.03). Participants reported a total of 292 medical events over 12 months; none were study‐related adverse events. A higher proportion of self‐help participants reported at least one exercise‐related medical event (16% vs. 5%; *p* = 0.003) or a new diagnosis, treatment, or hospitalization for depression (10% vs. 2%; *p* = 0.01) relative to the intervention group.

During months 7–12 (182 days), intervention participants tracked activity on a median (IQR) of 161 (52.5, 180.0) or 88.5% of days, which was significantly higher than self‐help participants (119.5 [11.5, 177.0], 65.7%) (*p* = 0.03). Engagement within the respective Facebook groups (i.e., ≥1 comment posted, viewed, or reacted to) was also significantly higher among intervention participants during maintenance (intervention: median [IQR] 22 (of 26) weeks [10.5, 25.0], 84.6% vs. self‐help: 10 [7.0, 13.5], 38.5%; *p* < 0.0001). Intervention participants could access weekly goal setting and bimonthly lessons and tailored feedback on the study website and had ≥1 login during a median (IQR) of 11 (2.0, 22.0) or 42.3% of weeks. Average accelerometer waking wear was comparable between groups at all assessments (baseline: 15.4 h/day (intervention), 15.5 h/day (self‐help); 6 months: 15.5 h/day (intervention, self‐help); 12 months: 15.2 h/day (intervention, self‐help)).

### 3.2 Total physical activity

**FIGURE 2:**
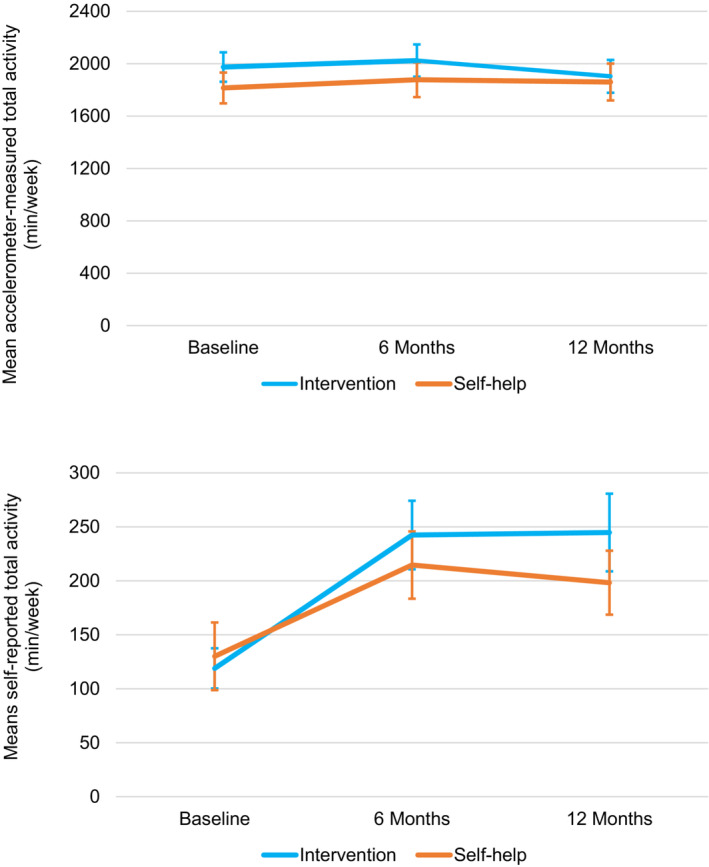
Changes in accelerometer‐measured and self‐reported total physical activity by group over 12 months. Unadjusted means, with error bars showing 95% confidence intervals. *N* in each group: 6‐month sample: accelerometer *n* = 122 (intervention), *n* = 129 (self‐help); self‐report *n* = 128 (intervention), *n* = 132 (self‐help); 12‐month sample: accelerometer *n* = 117 (intervention), *n* = 119 (self‐help); self‐report *n* = 121 (intervention), *n* = 124 (self‐help).

Table 1 shows between‐ and within‐group changes in PA and sedentary behavior outcomes between baseline to 12 months and 6 to 12 months. There was no between‐group difference (*p* = 0.25) or within‐group changes in accelerometer‐measured total PA from baseline to 12 months (*p*s = 0.32–0.57) (Figure 2). There was a significant intervention effect from baseline to 12 months in self‐reported total PA; increases were + 125.7 min/week (95% CI, 90.1–161.3) in the intervention versus +69.9 min/week (95% CI, 35.2–104.7) in the self‐help group (between‐group difference: 55.8 min/week, 95% CI, 6.0–105.6; *p* = 0.028). From 6 to 12 months, there were no between‐ or within‐group changes in accelerometer‐measured total PA (*p*s = 0.08–0.68) or self‐reported total PA (*p*s = 0.38–0.91). The correlation between accelerometer‐measured and self‐reported total PA at 12 months was significant (*ρ* = 0.1366, *p* = 0.036).

**TABLE 1:** Changes in physical activity and sedentary behavior within and between intervention and self‐help groups in the IMPACT Trial.

Outcome variable and group	Mean (SD)^a^			Within‐group change^b^ mean (95% CI)				Between‐group difference in change^b^ mean (95% CI)			
	Baseline	6 months	12 months	Baseline to 12 months	*p*	6–12 months	*p*	Baseline to 12 months	*p*	6–12 months	*p*
Accelerometer‐measured outcomes											
Total PA (min/week)								−92.9 (−252.1, 66.3)	0.25	−107.1 (−255.1, 41.0)	0.16
Intervention	1974.3 (673.9)	2024.3 (686.7)	1903.8 (683.5)	−27.8 (−124.5, 68.8)	0.57	−83.0 (−177.0, 11.1)	0.08				
Self‐help	1814.9 (704.5)	1877.7 (758.2)	1860.2 (774.6)	65.0 (−62.1, 192.2)	0.32	24.1 (−90.3, 138.5)	0.68				
Moderate‐to‐Vigorous PA (min/week)								8.5 (−8.8, 25.9)	0.34	−4.9 (−21.1, 11.3)	0.55
Intervention^c^	24.8 (31.2)	49.4 (59.9)^c^	45.9 (72.0)^d^	22.5 (8.8, 36.2)	0.001	−2.4 (−13.3, 8.6)	0.67				
Self‐help^c^	27.4 (34.4)	39.5 (57.1)^c^	41.5 (63.6)^d^	13.9 (3.0, 24.9)	0.013	2.5 (−9.6, 14.7)	0.68				
Light PA (min/week)								−102.7 (−259.4, 54.1)	0.20	−102.6 (−249.8, 44.5)	0.17
Intervention	1949.5 (664.6)	1974.9 (676.7)	1857.9 (678.2)	−50.8 (−144.7, 43.0)	0.29	−80.8 (−173.7, 12.1)	0.09				
Self‐help	1787.5 (695.6)	1838.2 (750.4)	1818.7 (753.5)	51.8 (−74.3, 178.0)	0.42	21.8 (−92.2, 135.8)	0.71				
Steps per day								−57.3 (−587.5, 472.8)	0.83	−434.3 (−913.5, 44.8)	0.08
Intervention^c^	8917.2 (2206)	9629.1 (2141)^c^	8956.2 (2464.0)	160.3 (−217.1, 537.6)	0.41	−515.4 (−840.5, −190.4)	0.002				
Self‐help	8394.5 (2116)	8779.3 (2344)	8538.7 (2562.5)	217.6 (−157.2, 592.4)	0.26	−81.1 (−435.0, 273.8)	0.65				
Sedentary behavior (min/day)								7.6 (−10.5, 25.6)	0.41	8.1 (−9.6, 25.8)	0.37
Intervention	546.2 (89.8)	538.0 (104.1)	543.9 (91.9)	5.3 (−6.7, 17.2)	0.39	12.9 (1.4, 24.4)	0.03				
Self‐help	557.4 (89.5)	551.0 (99.3)	546.3 (92.6)	−2.3 (−15.9, 11.3)	0.74	4.8 (−8.6, 18.2)	0.48				
Self‐report measured outcomes											
Total PA (min/week)								55.8 (6.0, 105.6)	0.028	15.7 (−33.6, 65.0)	0.53
Intervention^c^	118.9 (111.3)	242.5 (181.6)^c^	244.8 (200.0)^d^	125.7 (90.1, 161.3)	<0.0001	2.3 (−36.5, 41.1)	0.91				
Self‐help ^c^	130.1 (187.4)	214.6 (180.7)^c^	198.3 (166.7)^d^	69.9 (35.2, 104.7)	<0.0001	−13.4 (−43.5, 16.8)	0.38				
Moderate‐to‐vigorous PA (min/week)								29.9 (−0.7, 60.4)	0.055	24.2 (−6.3, 54.6)	0.12
Intervention^c^	56.6 (66.9)	134.3 (113.2)^c^	145.7 (129.7)^d^	88.6 (64.7, 112.4)	<0.0001	11.4 (−13.8, 36.6)	0.37				
Self‐help ^c^	51.7 (75.4)	123.8 (111.2)^c^	109.2 (104.9)^d^	58.7 (39.7, 77.7)	<0.0001	−12.8 (−29.8, 4.3)	0.14				
Light PA (min/week)								−11.6 (−90.5, 67.2)	0.77	−45.2 (−125.5, 35.2)	0.27
Intervention^c^	62.3 (73.5)	108.2 (126.5)^c^	99.1 (116.7)^d^	36.8 (16.6, 56.9)	0.0003	−9.4 (−35.9, 17.0)	0.48				
Self‐help	78.5 (153.9)	90.9 (107.7)	125.8 (421.9)	48.4 (−28.0, 124.8)	0.22	35.7 (−39.6, 111.1)	0.35				
Weekday sedentary behavior (min/day)								20.1 (−47.8, 87.9)	0.56	54.1 (−11.3, 119.4)	0.11
Intervention	638.1 (277.2)	628.4 (234.1)	666.1 (257.4)	17.9 (−31.9, 67.6)	0.48	37.5 (−7.6, 82.6)	0.10				
Self‐help	602.0 (255.5)	620.4 (244.3)	608.4 (225.2)	−2.2 (−48.1, 43.8)	0.93	−16.6 (−63.8, 30.7)	0.49				
Weekend sedentary behavior (min/day)								22.8 (−37.9, 83.5)	0.46	4.9 (−51.9, 61.7)	0.87
Intervention	507.5 (253.8)	494.2 (252.2)	523.9 (226.4)	8.6 (−32.7., 49.9)	0.68	27.4 (−12.3, 67.0)	0.18				
Self‐help	499.0 (247.0)	466.5 (214.5)	491.3 (217.5)	−14.2 (−58.5, 30.2)	0.53	22.5 (−18.2, 63.2)	0.28				

Abbreviations: CI, confidence interval; PA, physical activity; SD, standard deviation.

^a^ Unadjusted means. Baseline sample: *n* = 140 (intervention), *n* = 140 (self‐help); 6‐month sample: accelerometer *n* = 122 (intervention), *n* = 129 (self‐help); self‐report *n* = 128 (intervention), *n* = 132 (self‐help); 12‐month sample: accelerometer *n* = 117 (intervention), *n* = 119 (self‐help); self‐report *n* = 121 (intervention), *n* = 125 (self‐help).

^b^ Adjusted for education, time since diagnosis, age, and wear time (accelerometer only).

^c^ Within‐group improvement occurred between baseline and 6 months at *p* < 0.05 level of significance.

^d^ Within‐group improvement occurred between baseline and 12 months at *p* < 0.05 level of significance.

### 3.3 Moderate‐to‐vigorous physical activity (MVPA)

**FIGURE 3:**
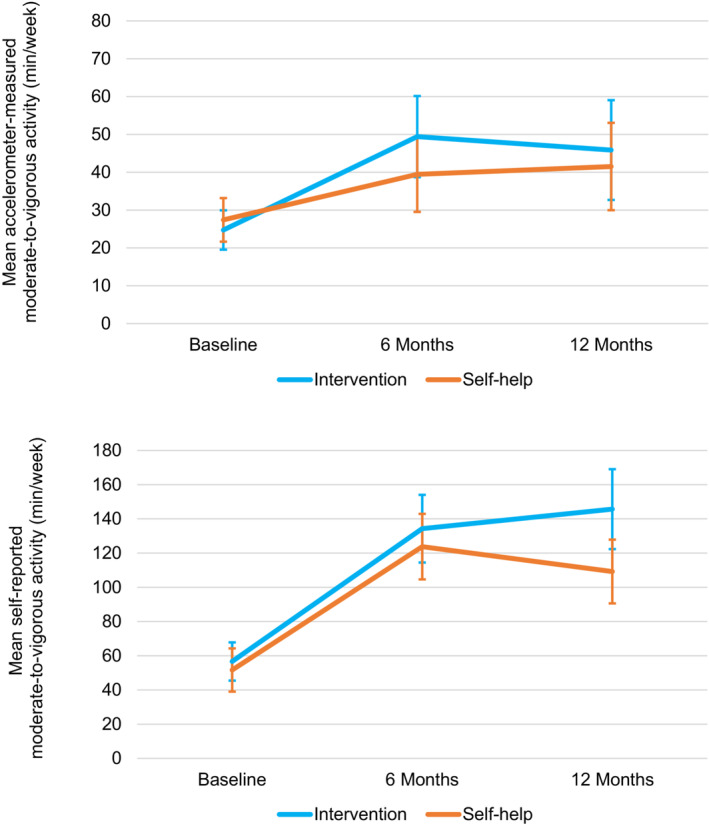
Changes in accelerometer‐measured and self‐reported moderate‐to‐vigorous physical activity by group over 12 months. Unadjusted means, with error bars showing 95% confidence intervals. *N* in each group: 6‐month sample: accelerometer *n* = 122 (intervention), *n* = 129 (self‐help); self‐report *n* = 128 (intervention), *n* = 132 (self‐help); 12‐month sample: accelerometer *n* = 117 (intervention), *n* = 119 (self‐help); self‐report *n* = 121 (intervention), *n* = 124 (self‐help).

Over 12 months, both groups increased accelerometer‐measured MVPA (+22.5 min/week; 95% CI, 8.76–36.2 in intervention vs. +13.9 min/week, 95% CI, 2.97–24.9 in self‐help), with no between‐group difference (*p* = 0.335) (Figure 3). Self‐reported MVPA also increased from baseline to 12 months within both groups (between‐group difference: 29.9 [95% CI, −0.67 to 60.4]; *p* = 0.055). Increases in accelerometer‐measured and self‐reported MVPA at 6 months were maintained at 12 months in both groups (*p*s = 0.14–0.68), with no between‐group differences (*p*s = 0.12–0.55). At 12 months, accelerometer‐measured and self‐reported MVPA were significantly correlated (*ρ* = 0.3219, *p* < 0.0001).

The proportion of YACS meeting PA guidelines at 12 months based on accelerometer data was 8.5% in the intervention versus 5.0% in the self‐help group, with no differences in relative risk (1.76 [95% CI, 0.68–4.56]; *p* = 0.24). Based on self‐reported MVPA, intervention participants had 1.5 times higher probability of meeting guidelines than self‐help participants (47.9% vs. 33.1%; RR, 1.45 [95% CI, 1.06–1.98]; *p* = 0.02)

### 3.4 Light physical activity, steps, and sedentary behavior

From baseline to 12 months, there were no between‐ or within‐group differences in accelerometer‐measured light PA (*p*s = 0.20–0.42), whereas the intervention group reported an increase at 12 months (36.8 min/week) versus no increase in the self‐help group (between‐group *p* = 0.77). Both accelerometer‐measured and self‐reported light PA were maintained from 6 to 12 months in both groups (*p*s = 0.09–0.71), with no between‐group differences (*p*s = 0.17–0.27).

There were no between‐group (*p* = 0.41–0.83) or within‐groups differences in changes in accelerometer‐measured steps/day (*p*s = 0.26–0.41) or sedentary behavior (*p*s = 0.39–0.74) from baseline to 12 months. From 6 to 12 months, the intervention group decreased steps/day (−515.4, 95% CI, −840.5, −190.4) and increased sedentary behavior (+12.9 min/day, 95% CI, 1.4–24.4), with no between‐group differences (*p* = 0.08–0.37). Self‐reported measures of sedentary behavior indicated no between‐ or within‐group changes from baseline to 12 months (*p*s = 0.46–0.93) or 6 to 12 months (*p*s = 0.10–0.87).

Analyses with participants who completed measures at all time points yielded similar results. When removing outliers in models of accelerometer‐measured total PA, between‐group differences in change from 6 to 12 months became significant favoring the self‐help group (−175.6 min/week, 95% CI, −308.9, −42.3; *p* = 0.01). Between‐group differences in changes in self‐reported MVPA from 6 to 12 months also became significant with outliers removed, such that increases were greater in the intervention than the self‐help group (+28.6 min/week, 95% CI, 4.2–53.1; *p* = 0.02).

## 4 DISCUSSION

While PA is well‐recognized as beneficial for cancer survivors, little research has examined longer‐term PA change and maintenance among YACS. The IMPACT intervention offered a mobile PA intervention to YACS who are at risk for adverse effects from cancer decades following their treatment. Following a 6‐month active intervention, tapered contacts and access to a moderated Facebook group did not result in greater increases in accelerometer‐measured total PA min/week in the intervention group at 12 months relative to a self‐help group. However, change in self‐reported total PA min/week from baseline to 12 months was significantly greater in the intervention versus self‐help group. Among both groups, levels of accelerometer‐ and self‐reported total PA were maintained between 6 and 12 months. Further, both groups improved accelerometer‐measured and self‐reported MVPA over 12 months and maintained improvements from 6 to 12 months. Intervention participants (48%) were more likely to report meeting PA guidelines than self‐help participants (33%) at 12 months. Overall, both digital approaches could be promising for promoting sustained PA participation and long‐term health benefits in YACS, but additional research is needed to identify what strategies work for whom, and under what conditions.

The lack of intervention effect on accelerometer‐measured total PA at 12 months is similar to prior studies with cancer survivors, which observed PA improvements in both intervention and control groups, resulting in a lack of effects.^39^, ^40^ Contamination by control group participants (i.e., adoption of PA) is common in PA trials in oncology, as participants may be motivated to change behavior and increase their PA regardless of group assignment (e.g., enrolling may increase the salience of PA and its benefits in addition to providing some relevant information and tools).^19^, ^41^ Indeed, self‐help participants had ongoing access to a widely available activity tracker and companion app and a Facebook group that facilitated use of behavior change techniques (e.g., self‐monitoring of behavior, goal setting, and social support) throughout the trial period. It is possible that the dose of theory‐based intervention enhancements wrapped around these tools was insufficient to significantly increase PA for some YACS, while for others only minimal intervention (self‐help condition) was needed to support adoption of PA. Additional research to identify moderators of intervention effects and characteristics of those who could potentially benefit from minimal intervention is warranted. In previous exercise oncology studies, contamination and dropout rates were lowest when control groups received an intervention both during and after the study intervention period.^41^ Future trials should carefully consider the design of comparison groups in trials of digital interventions to minimize bias.

Other home or web‐based interventions among survivors have reported significant intervention effects on PA at 12 months, but were based on self‐reported outcomes.^42^, ^43^, ^44^ Similarly, we found a significant intervention effect on self‐reported total PA at 12 months. While accelerometer and self‐reported PA measures were significantly correlated, the discrepancy between them is common among cancer survivors^20^, ^45^ and highlights the potential inaccuracy of participant perceptions of PA engagement or that accelerometry may not detect certain types of activities reported (e.g., cycling). All participants received Fitbit trackers which may have supported more accurate PA reporting. The present study's contribution was strengthened by collection of objectively measured PA, since few studies among survivors have reported PA maintenance using such measures.^19^, ^24^ Recent interventions incorporating activity trackers have examined PA maintenance following 3‐month interventions and found sustained effects at 3 months post‐intervention.^46^, ^47^, ^48^ More research is needed to understand the lack of agreement between self‐report, research‐grade accelerometry, and commercially available PA devices. Additionally, future work could validate PA measures with other measures of aerobic fitness and evaluate the effects of remotely delivered interventions using such measures.

At 12 months, there was no intervention effect on accelerometer‐measured MVPA, though intervention participants were more likely to report meeting MVPA guidelines than self‐help participants. The between‐group differences in self‐reported MVPA increases over 12 months approached significance. MVPA increases in the self‐help group were consistent with earlier definitions of contamination in home‐based trials (>60 min/week)^41^, ^49^ and may have contributed to underestimation of true intervention effects. Within‐group increases in self‐reported MVPA were higher (89 min/week intervention, 59 min/week self‐help) than those reported in a meta‐analysis of intervention studies evaluating long‐term PA change in cancer survivors ≥3 months post‐intervention (65 min/week intervention groups, 28 min/week control groups) and could be clinically meaningful.^19^ Of 19 studies reviewed, 35% observed significant MVPA improvements among control groups and only three reported accelerometer‐measured PA; Grimmett et al. concluded that low‐intensity interventions have the potential to support sustained PA behavior change among younger, motivated, well‐educated, and White survivors, which was the predominant composition of study samples and also with the current study.^19^ The sustained MVPA increase over time and maintenance from 6 to 12 months in both groups is notable and suggests that some YACS may benefit from digital tools and access to peer support alone, though this may be related to the study sample comprising participants that were predominantly female, well‐educated, and White. Similarly, a study among 80 breast cancer survivors found that access to an activity tracker alone supported maintenance of MVPA over 12 weeks following a 12‐week intervention with feedback, goal setting, and health coaching.^46^ On the contrary, another study among 59 survivors compared a PA maintenance intervention with Fitbit, text messages, and health coaching with a Fitbit‐only^50^ control, and found that the Fitbit alone was not sufficient to support MVPA maintenance after 8 weeks.^35^ Given the homogeneity of sample characteristics across these studies and emerging evidence that behavior changes after cancer diagnosis may vary among YACS by race and ethnicity,^51^ there is a need to evaluate PA interventions among more diverse samples of YACS. Adherence to PA guidelines has been shown to be lower among Black and Hispanic cancer survivors relative to those who are Non‐Hispanic White,^52^ and previous studies indicate that stronger PA intervention effects are observed when focused on specific subgroups of survivors with higher need.^53^ Future research should examine race and ethnicity as moderators of intervention effects and consider focusing on racially and ethnically diverse subgroups of YACS who could possibly benefit more from regular and sustained PA.

For some intervention participants, having continued access to goal setting prompted by text message, a Facebook group with moderated prompts, bimonthly lessons and tailored feedback (i.e., theory‐based intervention components designed to promote behavioral capability, self‐regulation, self‐efficacy, and social support) may have promoted successful PA maintenance from 6 to 12 months. Among cancer survivors, self‐regulatory (e.g., goal setting, self‐monitoring, behavioral feedback)^19^, ^47^, ^54^ and social support strategies appear to promote PA maintenance.^19^ The minimal between‐group differences may be attributable to both groups having ongoing exposure to these behavioral strategies through the digital tools that may have addressed barriers and facilitators of PA to varying degrees. All participants had ongoing access to an activity tracker and companion app that facilitated use of behavioral strategies (e.g., self‐monitoring of behavior, goal setting) throughout the trial period, and YACS have reported that goals help them maintain PA behaviors.^55^ Further, cancer survivors who use activity trackers to monitor activity or a health goal are more likely report meeting PA recommendations.^56^ Among YACS, lack of social support has been identified as a key barrier to PA adherence, while accountability to others is a facilitator.^55^ It is possible that the Facebook group was sufficient for some participants in both groups to enhance social support and accountability. The intervention was designed to address other reported PA facilitators (e.g., individualized, specific to YACS, setting goals) and intervention preferences among YACS (i.e., home‐based, choice, flexibility)^55^, ^57^, ^58^ by providing adaptive goals, feedback, and text messages that were individually tailored based on PA behavior and self‐reported characteristics (e.g., mood, fatigue). For some participants, the intervention dose or strategies used for theory‐based intervention enhancements may have been insufficient to address other common PA barriers among YACS such as fatigue, side effects, and negative emotions.^55^, ^57^, ^58^ The higher occurrence of exercise‐related injuries and new depression‐related events among the self‐help group lend support for a stepped care approach to support PA maintenance in cancer survivors.^19^ It is possible that in the absence of adaptive goals provided by the intervention, some self‐help participants attempted more rapid PA increases, raising their risk of injury. Given the heterogeneity of these physiological and psychosocial barriers among cancer survivors,^59^ interventions that more frequently adapt to YACS' individual behaviors and contexts are warranted. More work is needed to understand profiles of YACS who may be successful at adopting and maintaining PA using low‐intensity interventions, and under what contexts (e.g., cancer history and quality of life) YACS need additional types or exposures to behavior change techniques. Future research should consider utilizing the Multiphase Optimization Strategy (MOST) framework to optimize interventions that leverage the high use of digital tools in this population, such as just‐in‐time adaptive interventions, and provide PA intervention support, if and only when needed.

The decline in steps from 6 to 12 months within the intervention group may reflect decreases in light PA, which was also observed in a trial of PA maintenance following an intervention among survivors with activity trackers, group sessions, and a phone call.^47^ The increase in sedentary time in the intervention group during post‐intervention follow‐up is consistent with a trial among breast cancer survivors that evaluated PA maintenance following an intervention with activity trackers.^46^ There are documented health benefits of replacing sedentary behavior with either light PA or MVPA.^60^ While reducing risks associated with sedentary behaviors could be promoted in various ways (e.g., focusing on MVPA recommendations, reducing sedentary behavior without increasing MVPA),^61^, ^62^ the current intervention included limited content (i.e., one lesson, periodic tailored feedback and text messages) and did not provide specific sedentary behavior goals. Given the challenges in meeting MVPA recommendations and that increasing light PA may be more feasible for some cancer survivors,^8^ additional research should examine the relative benefits of reallocating sedentary time to light PA versus MVPA. Future interventions should optimize approaches to emphasizing PA of different intensities and identify effective combinations of strategies to reduce sedentary behaviors that are tailored to diverse individual contexts of cancer survivors. It has also been noted that underlying mechanisms or psychosocial determinants may vary between survivors who are inactive or somewhat active at baseline.^19^ Future research should clarify these mechanisms, identify predictors of PA maintenance in YACS (e.g., sociodemographic, clinical, and psychosocial), and develop effective strategies to more precisely tailor interventions to these predictors and individuals' previous PA experiences.

To our knowledge, this is the first study among YACS to evaluate digital intervention strategies to support PA adoption and maintenance over 12 months. Strengths of the study included the randomized controlled design, nationwide recruitment, remote delivery, strong retention, multiple measures of PA and sedentary behavior, and an active comparison group that facilitated evaluation of the sustained effects of access to digital tools and a Facebook group alone. Findings should also be considered in the context of limitations. The correlation between accelerometer‐ and self‐reported PA was significant but small, indicating bias in self‐reported PA measurement, which is reflected in the differing proportions of YACS meeting PA guidelines by measurement type. Since participants were not blinded to group assignment, intervention participants may have had higher social desirability bias to report higher levels of PA. Intervention participants received tapered contacts in months 7–12, which precluded the examination of intervention effects in the absence of continued contact. Given the relatively homogeneous study sample with respect to race, sex, and education, study findings may not be generalizable more broadly to YACS. Finally, the study focused on promotion of aerobic activity without emphasis on strength training, which is another component of the PA guidelines for cancer survivors.

This study contributes to the growing evidence that mHealth interventions with activity trackers may be effective for promoting and maintaining PA among cancer survivors. Findings suggest that a theory‐based intervention designed to enhance strategies offered by digital tools was more effective than a self‐help group at 12 months for self‐reported total PA and possibly self‐reported MVPA, but not accelerometer‐measured total PA or MVPA. Overall, both digital approaches have potential for dissemination to widely reach and promote PA maintenance and related health benefits among YACS. Additional research is needed to elucidate for whom, and under what contexts, specific intervention strategies work best.

## AUTHOR CONTRIBUTIONS

**Carmina G. Valle:** Conceptualization (lead); formal analysis (supporting); funding acquisition (lead); investigation (lead); methodology (lead); project administration (lead); supervision (lead); visualization (equal); writing – original draft (lead); writing – review and editing (lead). **Molly A. Diamond:** Investigation (equal); project administration (lead); validation (equal); writing – review and editing (equal). **Hillary Heiling:** Formal analysis (lead); methodology (equal); writing – original draft (supporting); writing – review and editing (equal). **Allison M. Deal:** Formal analysis (equal); methodology (equal); writing – original draft (supporting); writing – review and editing (equal). **Derek P. Hales:** Data curation (equal); formal analysis (equal); methodology (equal); validation (equal); writing – review and editing (equal). **Brooke T. Nezami:** Data curation (lead); formal analysis (supporting); investigation (equal); project administration (equal); validation (lead); writing – review and editing (equal). **Jessica Gokee LaRose:** Funding acquisition (supporting); methodology (supporting); writing – review and editing (equal). **Christine M. Rini:** Conceptualization (supporting); funding acquisition (equal); writing – review and editing (equal). **Bernardine M. Pinto:** Funding acquisition (equal); methodology (supporting); writing – review and editing (equal). **Deborah F. Tate:** Conceptualization (equal); funding acquisition (equal); methodology (equal); writing – review and editing (supporting).

## FUNDING INFORMATION

National Cancer Institute (R01CA204965; P30CA016086); National Institute of Diabetes and Digestive and Kidney Diseases (P30DK056350); University of North Carolina at Chapel Hill (University Cancer Research Fund); National Center for Advancing Translational Sciences (UL1TR002489).

## CONFLICT OF INTEREST STATEMENT

None.

## ETHICS APPROVAL

All procedures were approved by the University of North Carolina at Chapel Hill Oncology Protocol Review Committee (LCCC1707) and Institutional Review Board (#16‐3409) and performed in accordance with ethical standards of the 1964 Helsinki Declaration and its later amendments.

## CLINICALTRIALS.GOV IDENTIFIER

NCT03569605.

## Data Availability

The data that support the findings of this study are available from the corresponding author upon reasonable request. The data are not publicly available due to privacy or ethical restrictions.
